# Supplementary material for: Trends in the Use of Non-Pharmaceutical Interventions in Schools During the COVID-19 Pandemic, February 2021 to December 2023: A Mixed Methods Study
Source: Int J Environ Res Public Health. 2025 Mar 7;22(3):394. doi: 10.3390/ijerph22030394 (PMC11942009; doi:10.3390/ijerph22030394)
Supplement: Supplementary file 1 [file ijerph-22-00394-s001.zip › ijerph-3447929-supplementary-updated.pdf]

Supplementary material for *Trends in the use of non-pharmaceutical interventions in schools during the COVID-19 pandemic, February 2021 to December 2023: a mixed methods study*

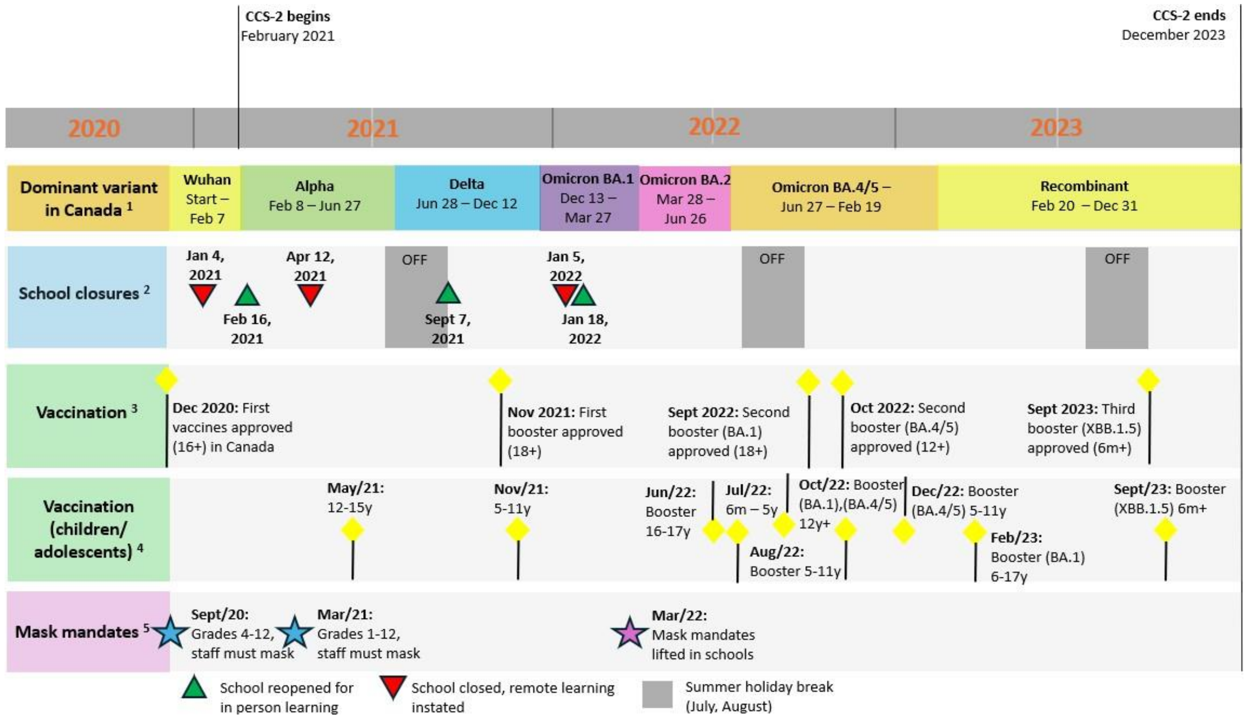

Supplementary Figure S1
Timeline of events during the COVID-19 Cohort Study for Teachers and Education Workers; Ontario Canada; February 2021-December 2023

CCS-2: COVID-19 Cohort Study for Teachers and Education Workers

84. Dominant variant in Canada: Government of Canada. COVID-19 epidemiology update: Current situation [Internet]. 2024 [cited 2024 Mar 5]. Available from: https://health-infobase.canada.ca/covid-19/current-situation.html.

85. School closures: Akanteva A, Dick DW, Amiraslani S, Heffernan JM. Canadian Covid-19 pandemic public health mitigation measures at the province level. Sci Data. 2023;10(1):882. doi:10.1038/s41597-023-02759-y

86. Vaccination: Health Canada. Health Canada authorizes first COVID-19 vaccine [Internet]. 2020 [cited 2024 Oct 1]. Available from: https://www.canada.ca/en/health-canada/news/2020/12/health-canada-authorizes-first-covid-19-vaccine0.html.

87. Health Canada. Health Canada authorizes the use of the Pfizer-BioNTech Comirnaty COVID-19 vaccine as a booster shot [Internet]. 2021 [cited 2024 Oct 1]. Available from: https://www.canada.ca/en/health-canada/news/2021/11/health-canada-authorizes-the-use-of-the-pfizer-biontech-comirnaty-covid-19-vaccine-as-a-booster-shot.html.

88. Health Canada. Health Canada authorizes first bivalent COVID-19 booster for adults 18 years and older [Internet]. Government of Canada; 2022 [cited 2024 Oct 1]. Available from: https://www.canada.ca/en/health-canada/news/2022/09/health-canada-authorizes-first-bivalent-covid-19-booster-for-adults-18-years-and-older.html.

89. Health Canada. Health Canada authorizes COVID-19 vaccine booster targeting the Omicron BA.4/BA.5 subvariants [Internet]. 2022 [cited 2024 Oct 1]. Available from: https://www.canada.ca/en/health-canada/news/2022/10/health-canada-authorizes- covid-19-vaccine-booster-targeting-the-omicron-ba4ba5-subvariants.html.

90. Health Canada. Health Canada authorizes Moderna COVID-19 vaccine targeting the Omicron XBB.1.5 subvariant [Internet]. 2023 [cited 2024 Oct 1]. Available from: https://www.canada.ca/en/health-canada/news/2023/09/health-canada-authorizes- moderna-covid-19-vaccine-targeting-the-omicron-xbb15-subvariant.html.

91. Vaccination (children/adolescents): Health Canada. Health Canada authorizes use of the Pfizer-BioNTech COVID-19 vaccine in children 12 to 15 years of age [Internet]. 2021 [cited 2024 Oct 18]. Available from: https://www.canada.ca/en/health-canada/news/2021/05/health-canada-authorizes-use-of-the-pfizer-biontech-covid-19-

vaccine-in-children-12-to-15-years-of-age.html.

92. Health Canada. Health Canada authorizes use of Comirnaty (the Pfizer-BioNTech COVID-19 vaccine) in children 5 to 11 years of age [Internet]. 2021 [cited 2024 Oct 18]. Available from: https://www.canada.ca/en/health-canada/news/2021/11/health- canada-authorizes-use-of-comirnaty-the-pfizer-biontech-covid-19-vaccine-in-children-5-to-11-years-of-age.html.

93. Health Canada. Health Canada authorizes use of Moderna COVID-19 vaccine in children 6 months to 5 years of age [Internet]. 2022 [cited 2024 Oct 18]. Available from: https://www.canada.ca/en/health-canada/news/2022/07/health-canada- authorizes-use-of-moderna-covid-19-vaccine-in-children-6-months-to-5-years-of-age.html.

94. Government of Canada. Regulatory Decision Summary - Comirnaty Original/Omicron BA.1 - Health Canada [Internet]. 2022 [cited 2024 Oct 18]. Available from: https://covid-vaccine.canada.ca/info/regulatory-decision-summary-detail.html?linkID=RDS01012.

95. Government of Canada. Regulatory Decision Summary - Comirnaty Original & Omicron BA.4/BA.5 - Health Canada [Internet]. 2022 [cited 2024 Oct 18]. Available from: https://covid-vaccine.canada.ca/info/regulatory-decision-summary-detail.html?linkID=RDS01006.

96. Government of Canada. Regulatory Decision Summary - Comirnaty Original & Omicron BA.4/BA.5 - Health Canada [Internet]. 2022 [cited 2024 Oct 18]. Available from: https://covid-vaccine.canada.ca/info/regulatory-decision-summary-detail.html?linkID=RDS01030.

97. Public Health Agency of Canada. Summary of NACI statement of August 19, 2022: Recommendations on the use of a first booster dose of Pfizer-BioNTech Comirnaty COVID-19 vaccine in children 5 to 11 years of age [Internet]. 2022 [cited 2024 Oct 18]. Available from: https://www.canada.ca/en/public-health/services/immunization/national-advisory-committee-on-immunization-naci/recommendations-use-first-booster-dose-pfizer-biontech-comirnaty-covid-19-vaccine-children-5-11-years/summary-august-19-2022.html.

98. Government of Canada. Regulatory Decision Summary - Spikevax - Health Canada [Internet]. 2023 [cited 2024 Oct 18]. Available from: https://covid-vaccine.canada.ca/info/regulatory-decision-summary-detail.html?linkID=RDS01052.

99. Government of Canada. Regulatory Decision Summary - Spikevax Bivalent - Health Canada [Internet]. 2023 [cited 2024 Oct 18]. Available from: https://covid-vaccine.canada.ca/info/regulatory-decision-summary-detail.html?linkID=RDS01076.

100. Government of Canada. Regulatory Decision Summary for Comirnaty Omicron XBB.1.5 [Internet]. 2023 [cited 2024 Oct 18]. Available from: https://covid-vaccine.canada.ca/info/RDS1695846822831-comirnaty-omicron-xbb-1-5-en.html.

101. Mask mandates: Ontario Ministry of Education. Guide to reopening Ontario's schools [Internet]. 2020 [cited 2020 Sept 1]. Available from: https://web.archive.org/web/20200901004807/https://www.ontario.ca/page/guide-reopening-ontarios-schools.

102. Ontario Ministry of Education. Guide to reopening Ontario's schools [Internet]. 2021 [cited 2021 Mar 3]. Available from: https://web.archive.org/web/20210303235742/https://www.ontario.ca/page/guide-reopening-ontarios-schools.

103. CBC News. (2022). *Ontario lifts mask mandates in most spaces, but it's no 'light switch' for pre-pandemic life, expert says*. https://www.cbc.ca/news/canada/toronto/covid19-ont-masks-march-21-2022-1.6385293

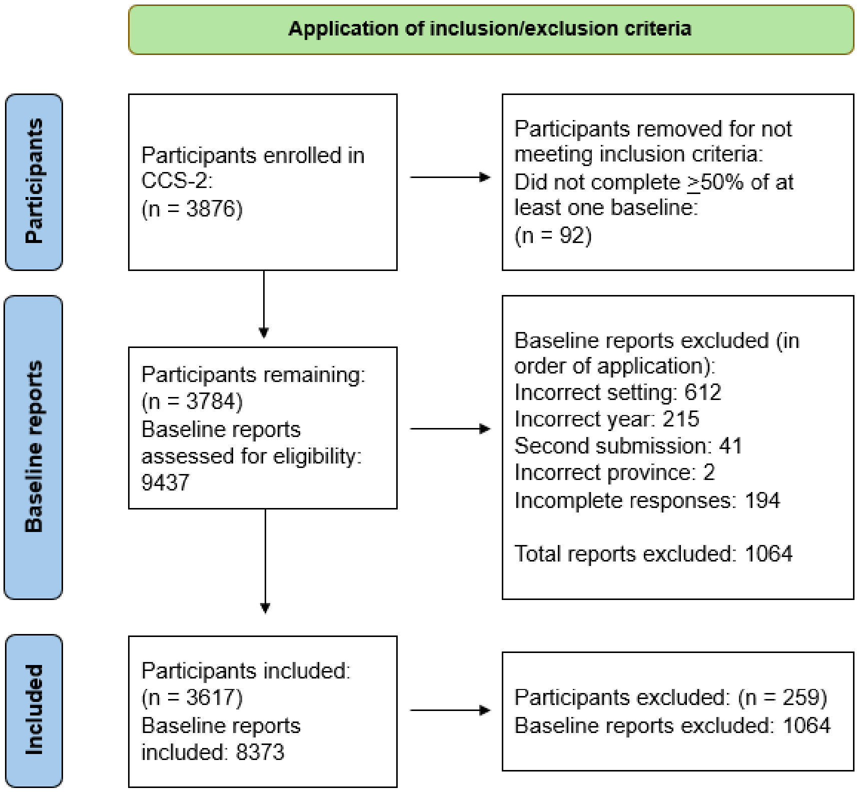

Supplementary Figure S2
Flow chart of participant and baseline report inclusion; COVID-19 Cohort Study for Teachers and Education Workers; Ontario Canada; February 2021-December 2023

Supplementary Table S1

Characteristics of Ontario education workers at time of eligible baseline submission, by half-year; COVID-19 Cohort Study for Teachers and Education Workers (February 2021-December 2023), Number (percent) unless otherwise noted

	Feb/21 - Aug/21	Sept/21 - Jan/22	Feb/22 - Aug/22	Sept/22 - Jan/23	Feb/23 - Aug/23	Sept/23- Dec/23
**Characteristic**	N = 2945	N = 1498	N = 703	N = 1494	N = 268	N = 1465
**Demographic characteristics**						
Age in years, mean (95% CI)	45.4 (45,46)	45.8 (45,46)	47.8 (47,48)	47.6 (47,48)	46.4 (45,47)	48.6 (48,49)
Gender						
Male	424 (14.4)	189 (12.6)	125 (17.8)	200 (13.4)	38 (14.2)	191 (13.1)
Female	2514 (85.6)	1307 (87.4)	576 (82.2)	1290 (86.6)	229 (85.7)	1269 (86.9)
Other / Non-binary	7 (0.2)	2 (0.1)	2 (0.3)	4 (0.3)	1 (0.4)	5 (0.3)
Education, highest achieved						
College diploma or less	284 (9.6)	115 (7.7)	53 (7.5)	119 (8.0)	15 (5.6)	110 (7.5)
Bachelor's degree/teaching certification	1980 (67.2)	1032 (68.9)	480 (68.3)	1017 (68.1)	199 (74.3)	1025 (70.0)
Master's degree/PhD	681 (23.1)	351 (23.4)	170 (24.2)	358 (24.0)	54 (20.1)	330 (22.5)
Occupation						
Teacher	2371 (80.5)	1238 (82.6)	566 (80.5)	1219 (81.6)	231 (86.2)	1222 (83.4)
Educational assistant	188 (6.4)	75 (5.0)	42 (6.0)	101 (6.8)	11 (4.1)	89 (6.1)
Early childhood educator	65 (2.2)	39 (2.6)	14 (2.0)	25 (1.7)	6 (2.2)	22 (1.5)
Principal / vice principal	117 (4.0)	48 (3.2)	39 (5.6)	58 (3.9)	7 (2.6)	50 (3.4)
Administration[1]	71 (2.4)	29 (1.9)	19 (2.7)	36 (2.4)	4 (1.5)	32 (2.2)
Professional student services roles[2]	103 (3.5)	53 (3.5)	18 (2.6)	44 (3.0)	8 (3.0)	42 (2.9)
Support staff[3]	30 (1.0)	16 (1.1)	5 (0.7)	11 (0.7)	1 (0.4)	8 (0.6)
Postal district						
Eastern Ontario	536 (18.2)	269 (18.0)	124 (17.6)	275 (18.4)	56 (20.9)	278 (19.0)
Central Ontario	956 (32.5)	545 (36.4)	236 (33.6)	531 (35.5)	97 (36.2)	526 (35.9)
Metropolitan Toronto	598 (20.3)	289 (19.3)	139 (19.8)	299 (20.0)	46 (17.2)	279 (19.0)
Southwestern Ontario	714 (24.2)	329 (22.0)	176 (25.0)	323 (21.6)	56 (20.9)	314 (21.4)
Northern Ontario	141 (4.8)	66 (4.4)	28 (4.0)	66 (4.4)	13 (4.9)	68 (4.7)
**Health related characteristics**						
Chronic illness[4] (vs no)	722 (24.5)	367 (24.5)	174 (24.7)	368 (24.6)	75 (28.0)	384 (26.2)
COVID-19 prior to questionnaire (vs no)	70 (2.4)	44 (2.9)	234 (33.3)	759 (50.8)	160 (59.7)	1025 (70.0)
**School characteristics**						
Size of school						
No specific school	50 (1.7)	27 (1.8)	3 (0.4)	24 (1.6)	5 (1.9)	22 (1.5)
<400	1160 (39.3)	484 (32.3)	222 (31.6)	459 (30.7)	72 (26.9)	427 (29.1)
400-699	930 (31.6)	489 (32.6)	228 (32.4)	486 (32.5)	86 (32.1)	481 (32.8)
700+	805 (27.3)	498 (33.2)	250 (35.6)	525 (35.1)	105 (39.2)	535 (36.5)
Highest reported contact level with students						
No close contact	78 (2.6)	23 (1.5)	19 (2.7)	17 (1.1)	4 (1.5)	9 (0.6)
Same room, >2 metres	515 (17.5)	125 (8.3)	56 (8.0)	75 (5.0)	11 (4.1)	65 (4.4)
Same room, <2 metres	1611 (54.7)	958 (64.0)	416 (59.2)	953 (63.8)	166 (61.9)	901 (61.5)
Physical contact	741 (25.2)	392 (26.2)	212 (30.2)	449 (30.1)	87 (32.5)	490 (33.5)

CI: confidence interval

[1] Office/clerical staff, superintendents

[2] Psychologist, social worker, therapist, librarian, nurse

[3] Technicians, bus drivers, custodians, building maintenance, cafeteria staff, lunchroom assistant

[4] Asthma, chronic obstructive pulmonary disease or other chronic lung condition, diabetes, heart disease, cancer treated in the past five years, liver or kidney disease, HIV/AIDS or other immune suppressing disease/condition, chronic neurological disorder, or other long-term chronic conditions

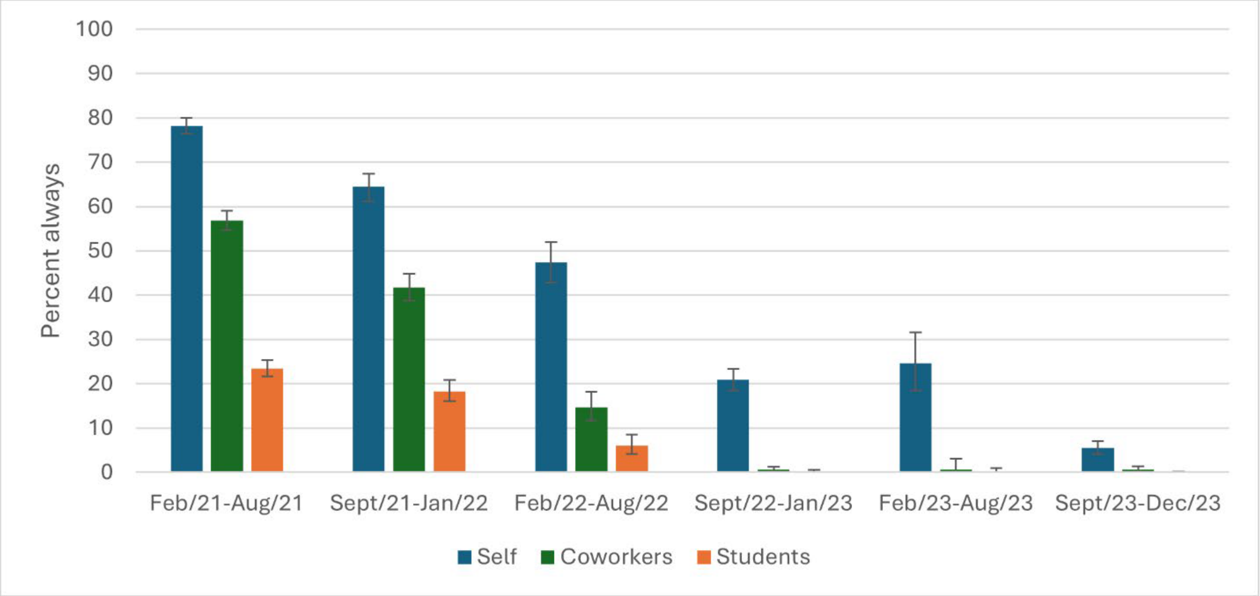

Supplementary Figure S3
Wears a mask in others' presence while at work as reported by Ontario elementary school education workers for themselves, their coworkers, and their students; COVID-19 Cohort Study for Teachers and Education Workers (February 2021 to December 2023). Vertical bars indicate 95% confidence intervals

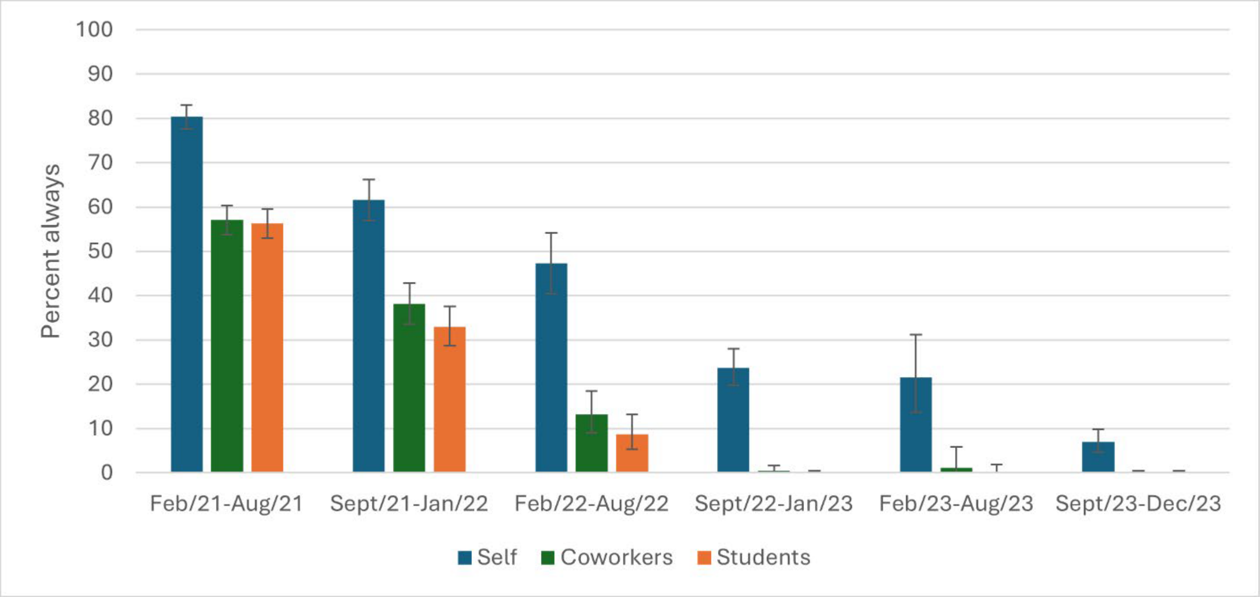

Supplementary Figure S4
Wears a mask in others' presence while at work as reported by Ontario secondary school education workers for themselves, their coworkers, and their students; COVID-19 Cohort Study for Teachers and Education Workers (February 2021 to December 2023). Bars indicate 95% confidence intervals

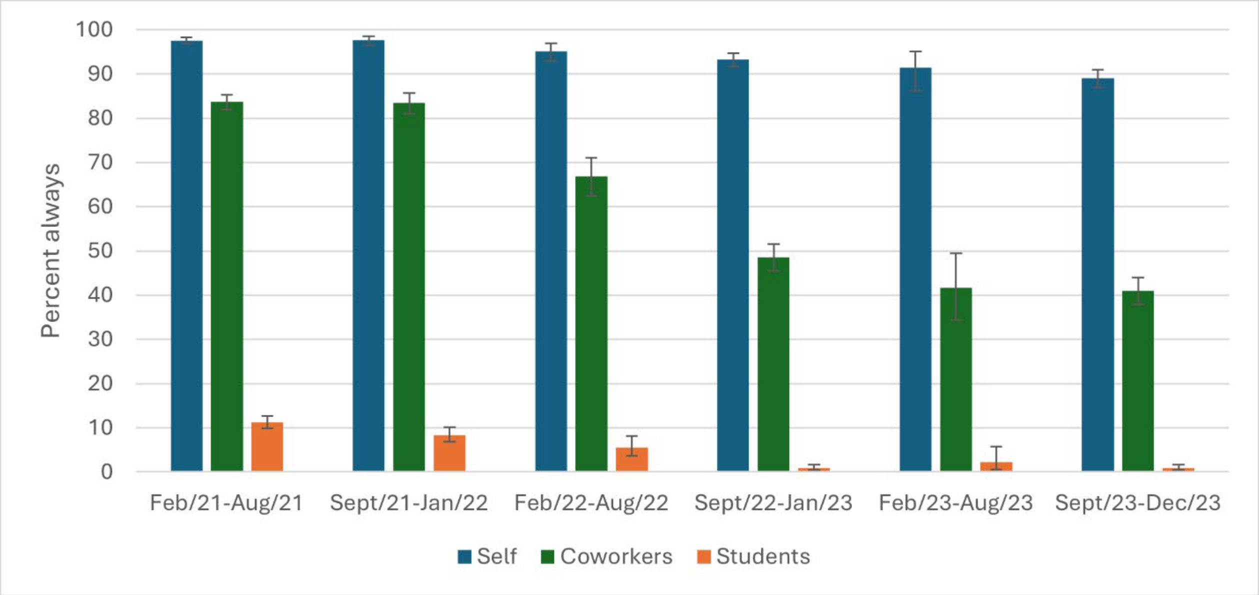

Supplementary Figure S5
Covers coughs while at work as reported by Ontario elementary school education workers for themselves, their coworkers, and their students; COVID-19 Cohort Study for Teachers and Education Workers (February 2021 to December 2023). Bars indicate 95% confidence intervals

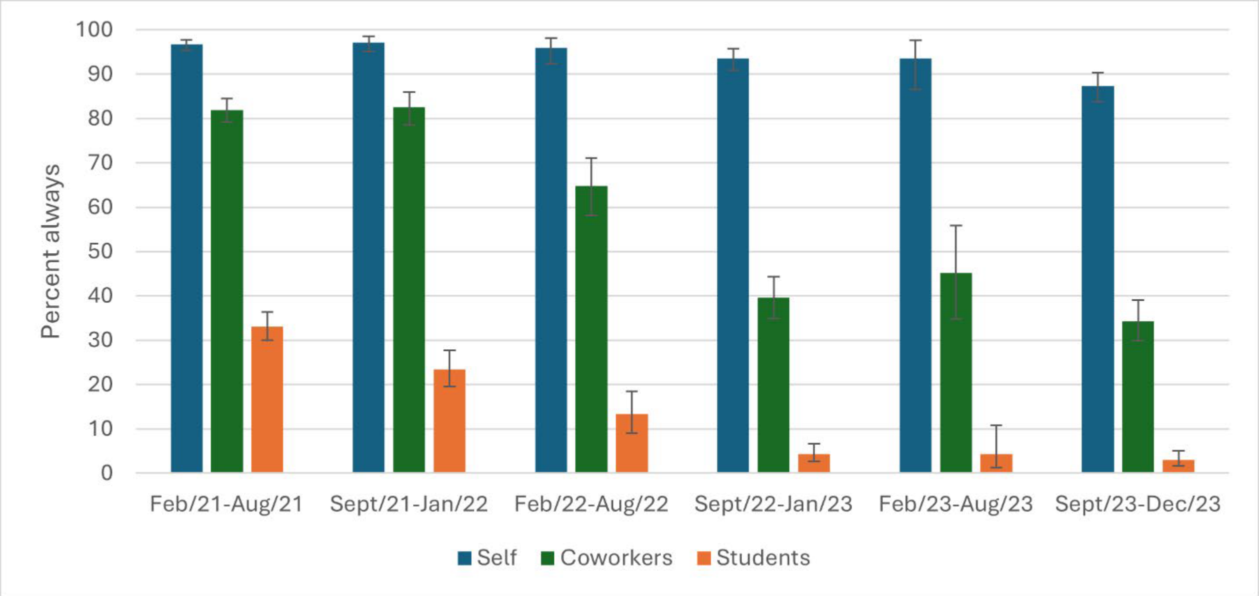

Supplementary Figure S6
Covers coughs while at work as reported by Ontario secondary school education workers for themselves, their coworkers, and their students; COVID-19 Cohort Study for Teachers and Education Workers (February 2021 to December 2023). Bars indicate 95% confidence intervals

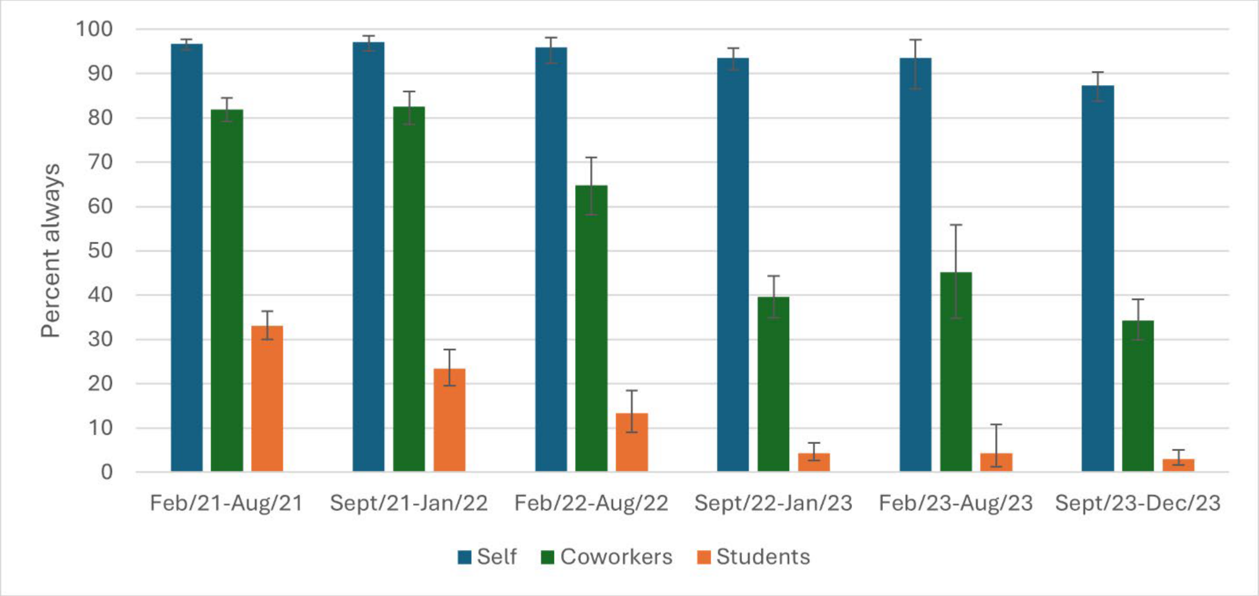

Supplementary Figure S7
Washes hands thoroughly and regularly while at work as reported by Ontario elementary school education workers for themselves, their coworkers, and their students; COVID-19 Cohort Study for Teachers and Education Workers (February 2021 to December 2023). Bars indicate 95% confidence intervals

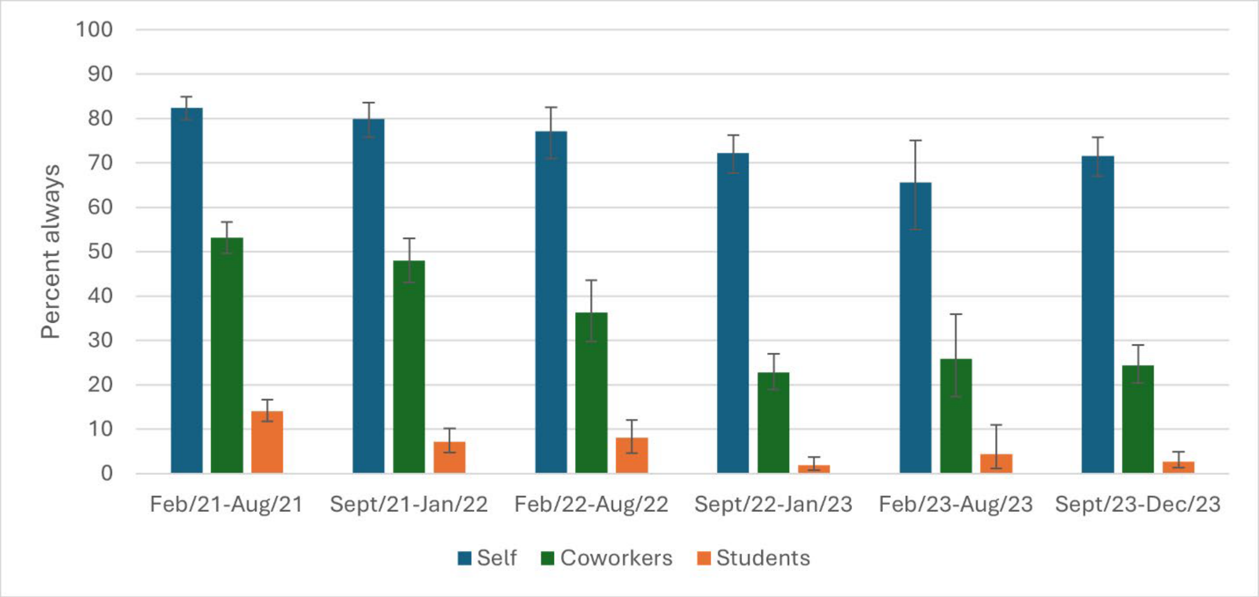

Supplementary Figure S8
Washes hands thoroughly and regularly while at work as reported by Ontario secondary school education workers for themselves, their coworkers, and their students; COVID-19 Cohort Study for Teachers and Education Workers (February 2021 to December 2023). Bars indicate 95% confidence intervals

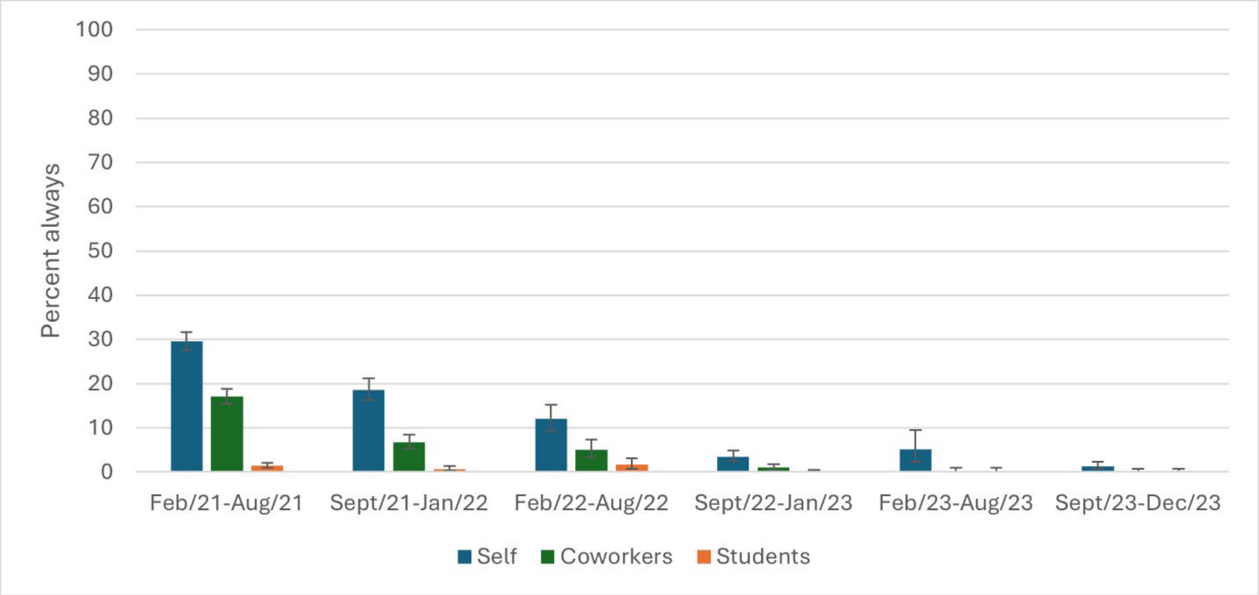

Supplementary Figure S9
Physically distances* from others while at work as reported by Ontario elementary school education workers for themselves, their coworkers, and their students; COVID-19 Cohort Study for Teachers and Education Workers (February 2021 to December 2023). Bars indicate 95% confidence intervals
***A specific distance was not included in the question**

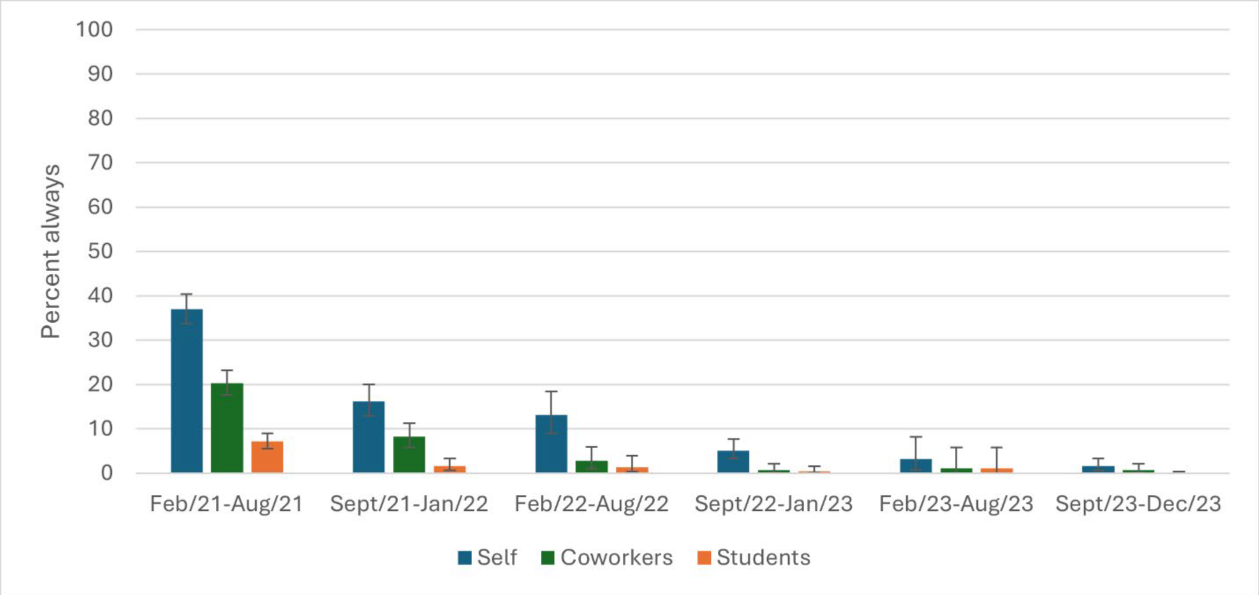

Supplementary Figure S10
Physically distances* from others while at work as reported by Ontario secondary school education workers for themselves, their coworkers, and their students; COVID-19 Cohort Study for Teachers and Education Workers (February 2021 to December 2023). Bars indicate 95% confidence intervals
***A specific distance was not included in the question**

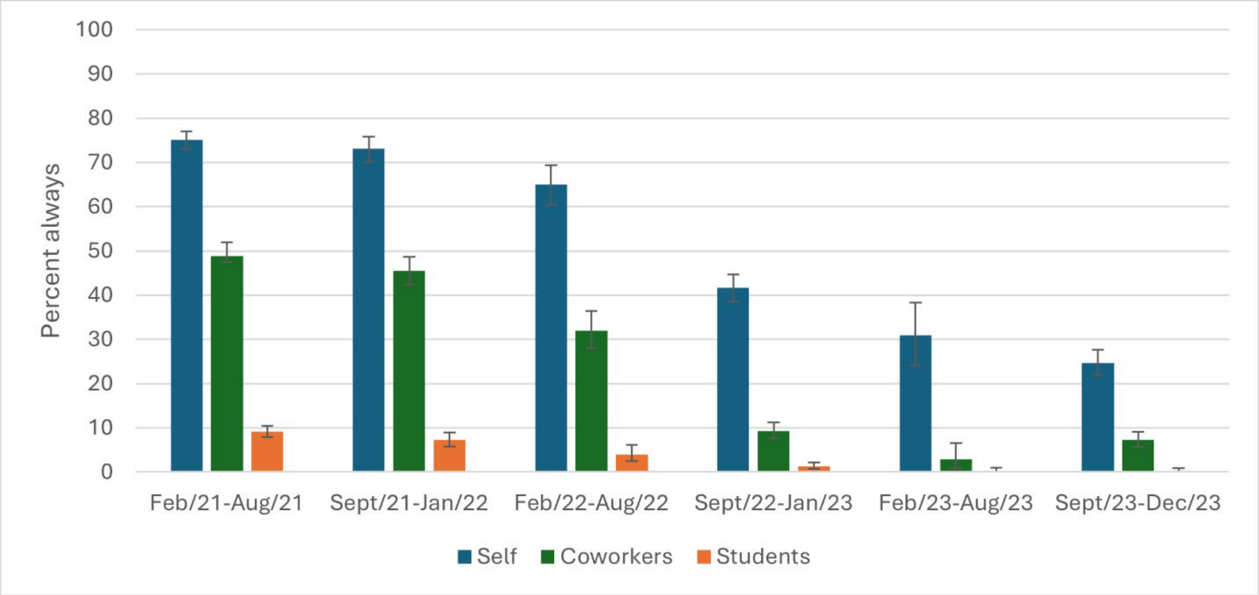

Supplementary Figure S11
Stays home when they have symptoms, even if they are mild as reported by Ontario elementary school education workers for themselves, their coworkers, and their students; COVID-19 Cohort Study for Teachers and Education Workers (February 2021 to December 2023). Bars indicate 95% confidence intervals

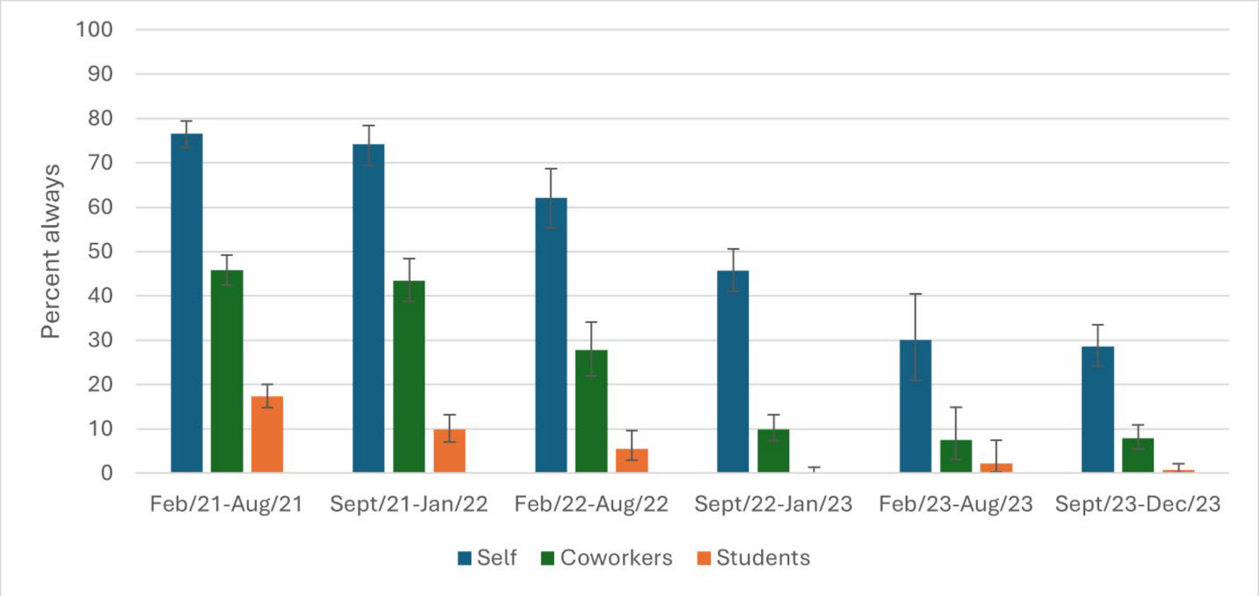

Supplementary Figure S12
Stays home when they have symptoms, even if they are mild as reported by Ontario secondary school education workers for themselves, their coworkers, and their students; COVID-19 Cohort Study for Teachers and Education Workers (February 2021 to December 2023). Bars indicate 95% confidence intervals
